# Laparoscopic and Endoscopic Cooperative Dissection for Small Gastric Gastrointestinal Stromal Tumor without Causing Injury to the Mucosa

**DOI:** 10.1155/2019/7376903

**Published:** 2019-12-13

**Authors:** Lei Cao, Kunming Zheng, Honglei Wang, Yongjie Zhao, Zhengduo Yang, Wen Li

**Affiliations:** ^1^Department of General Surgery, Tianjin Union Medical Center, Tianjin 300121, China; ^2^Department of Pathology, Tianjin Union Medical Center, Tianjin 300121, China; ^3^Endoscopic Center, Tianjin Union Medical Center, China

## Abstract

**Objective:**

To investigate the feasibility of laparoscopic and endoscopic cooperative dissection (LECD) for small gastric gastrointestinal stromal tumors (GISTs) without causing injury to the mucosa, compared with ESD surgery which is widely used now.

**Methods:**

A total of 25 patients with small gastric GISTs who underwent LECD and 20 patients with small gastric GISTs who underwent ESD between October 2014 and June 2016 were included in this study. All patients underwent curative resection for pathologically diagnosed small gastric GISTs. Patients' clinical data were retrospectively analyzed.

**Results:**

In LECD group, the operation was successfully performed in all patients. However, in the ESD group, three patients were transferred to laparoscopic surgery due to intraoperative massive bleeding or intraoperative perforation. No additional targeted chemotherapy drugs for interstitial tumors were prescribed in two groups. There was no difference in the complete tumor capsule rate (100% vs. 90%, *p* = 0.11), operation time (80.76 ± 13.86 ml vs. 84.05 ± 15.33 ml, *p* = 0.45), major intraoperative bleeding (0 vs. 5%, *p* = 0.26), postoperative bleeding (0 vs. 10%, *p* = 0.11), and postoperative infection (0 vs. 10%, *p* = 0.11) between the two groups. Compared to ESD (endoscopic submucosal dissection), LECS patients had shorter postoperative indwelling gastric tube (1.04 ± 0.98 d vs. 2.85 ± 0.24 d, *p* < 0.01), earlier postoperative eating (1.96 ± 0.98 d vs. 3.50 ± 1.15 d, *p* < 0.01), shorter average postoperative hospital stay (3.44 ± 1.00 d vs. 7.85 ± 1.18 d, *p* < 0.01), smaller perforation rate (0 vs. 25%, *p* < 0.05), and fewer surgical supplies. No recurrence or metastasis cases were found between the two groups during the follow-up period, and there were no cases of death due to gastric GISTs.

**Conclusion:**

LECD is a novel surgery for small gastric gastrointestinal stromal tumors that leads to satisfactory short-term outcomes and meets the idea of minimally invasive surgery and rapid recovery; compared with ESD, LECD surgery has some advantages in clinical practice. However, further follow-up is needed to confirm.

## 1. Introduction

Gastrointestinal stromal tumor (GIST) is the most common mesenchymal neoplasm that can be found in any part of the digestive system; the most common sites are the stomach and small intestine. The annual incidence of GIST ranges between 1 and 2 per 100,000, and 20%~35% of these are small GISTs (tumor with less than 2 cm in diameter) [1, 2]. At present, surgical resection remains the main therapeutic approach for nonmetastatic GISTs [3, 4]. Currently, laparoscopic and endoscopic cooperative wedge resection has been widely accepted and is now considered to be a minimally invasive surgery for GISTs in the stomach. However, wedge resection often causes damage to gastric mucosa. Over recent years, the studies on the surgical methods for gastric interstitial tumors have mainly focused on minimally invasive surgery and rapid recovery.

We herein propose a new surgical approach for the small gastric GISTs, laparoscopic and endoscopic cooperative dissection (LECD) for small gastric gastrointestinal stromal tumors without injury the mucosa, and achieved good therapeutic results. Meanwhile, compared with ESD surgery which is widely used now, LECD surgery has some advantages in clinical practice.

## 2. Patients and Methods

### 2.1. Patients

A total of 25 patients with small gastric GISTs who underwent LECD and 20 patients with small gastric GISTs who underwent ESD between October 2014 and June 2016 were included in this study. In LECD group, there were 13 male and 12 female patients, the mean patient age was 60.12 ± 9.21 years, and the average Body Mass Index (BMI) was 22.52 ± 2.41 kg/m^2^. In ESD group, there were 9 male and 11 female patients, mean patient age was 58.25 ± 9.86 years, and the average Body Mass Index (BMI) was 22.46 ± 2.36 kg/m^2^. The inclusion criteria for patients in the ESD group were consistent with those in the LECD group. The inclusion criteria were the following: ① Preoperative endoscopic ultrasonography indicated that the tumor was originated from the intrinsic muscular layer (from the 4^th^ layer of the wall of the stomach), and the tumor was first considered to be gastric stromal tumor. ② Tumor diameter is less than 2 cm. ③ Preoperative imaging examination showed no partial and distant metastasis. ④ Preoperative cardiopulmonary function and other basic state assessment were favorable, and no general anesthesia contraindications were observed. ⑤ Patent had no complicated surgical history and severe abdominal adhesion.

The institutional ethics review board of our hospital approved the protocol, and informed consent was obtained from all the patients.

### 2.2. Procedure

**Figure 1:**
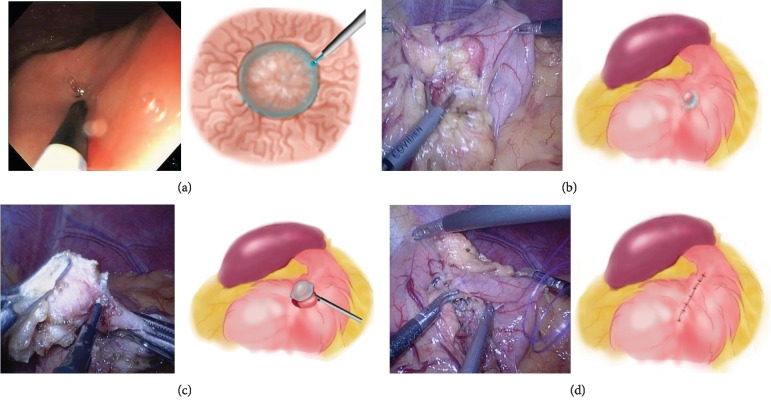
Real-time images and schematic procedure of laparoscopic and endoscopic cooperative dissection (LECD) for small GISTs. (a) Indigo carmine dye was endoscopically injected into the submucosal layer. (b) The position of the tumor was observed under laparoscopy. (c) The gastric serous layer was cut open; the tumor was dissected using image-guided laparoscope. (d) To suture the incision of gastric serosa under the laparoscope.

In LECD procedure, the patient was placed in the supine position with the legs spread apart. The brief operation flow is shown in schematic view (Figure 1). Briefly, a laparoscopy was preferred on the umbilicus. The intragastric location of the GIST was then confirmed by the intraoperative endoscopy, and the serosal position of the tumor was determined under the image-guided laparoscope by pressing the gastric wall using the tip of the endoscopy. The submucosa was then punctured; a small amount of indigo carmine dye was endoscopically injected into the submucosal layer circumferentially, separating stromal tumor and mucous membrane separation; and finally, the position of the tumor in the serous membrane was identified under the laparoscope. Consequently, the gastric wall was cut open in serous layer using electrotome under the laparoscope, and the tumor was dissected. During this process, it is important to protect the integrity of the mucosal layer and avoid the occurrence of mucosal layer damage. Finally, the incision of gastric serosa was sutured using absorb string under the laparoscope.

**Figure 2:**
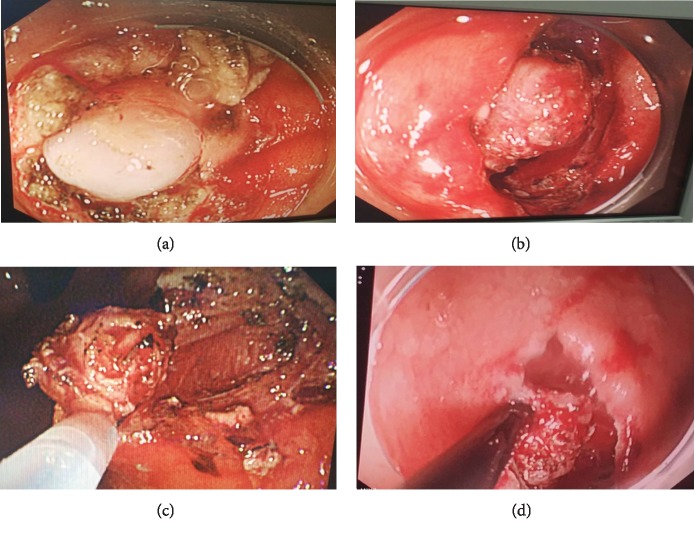
Real-time image procedure of endoscopic submucosal dissection (ESD) for small GISTs. (a, b) Endoscopic exposure of gastric stromal tumor. (c) Endoscopic removal of gastric stromal tumor. (d) Perforation during ESD.

In ESD procedure (Figure 2), after the lesion boundary was identified, markers were set at a distance of approximately 5 mm around the boundary. Submucosal injections of a mixture of saline solution, glycerol fructose, and sodium hyaluronate were administered, followed by a circumferential mucosal incision outside the markers with a hook-type knife. After the incision was made and an additional submucosal injection was administered, the submucosal layer was dissected using a hook-type knife, and the tumor was further dissected after being exposed. Bleeding and perforations occurring during ESDs were treated as follows: A hemostatic forceps was used to stop the bleeding, and endoscopic clips were used to close the perforation. Laparoscopic surgery was used for uncontrolled bleeding and perforation in patients in whom endoscopic treatment failed to resolve the problem.

### 2.3. Follow-Up Visit

Follow-up methods included phone calls, postoperative gastroscopy, and CT examination. The content of the follow-up included patient survival condition, postoperative follow-up review results, tumor recurrence, and/or metastasis. The end of the follow-up time was March 2018.

### 2.4. Statistical Analysis

The statistical analyses were performed using the SPSS 17.0 software. Parametric data were presented as mean ± SEM, and differences between each group were analyzed using Student's *t*-test. The clinicopathological parameters was evaluated by the *χ*^2^ test or Fisher's exact test when appropriate. All of the *p* values reported were two-sided, and significance was defined as *p* < 0.05.

## 3. Results

### 3.1. Preoperation Results

In LECD groups, all the 25 lesions were successfully resected under the laparoscope and endoscopy. There was no conversion to open surgery and no intraoperative mucous layer perforations. Tumor locations were explored by intraoperative gastroscopy and laparoscopy. In 11 cases (44.0%), the tumor was found in the fundus of the stomach; in 10 cases (40.0%), in the body of the stomach; and in 4 cases (16.0%), in the antrum of the stomach. The mean diameter of the tumor was 1.38 ± 0.5 cm. The mean operation time and blood loss were 80.76 ± 13.86 min and 21.60 ± 13.23 ml, respectively. The average postoperative hospital stay was 3.44 ± 1.00 d. None of the patients received abdominal drainage tube, and the gastric tube was pulled out on the day or day after of the operation in most patients. Postoperative time to recovery of liquid diet was 1.96 ± 0.98 d. In addition, none of the patients had surgical complications, and no targeted chemotherapy drugs for interstitial tumors were prescribed.

We compared the cases of LECD with endoscopic submucosal dissection (ESD) for the treatment of small gastric GISTs in our hospital at the same period. The inclusion criteria for patients in the ESD group were consistent with those in the LECD group. For statistical comparisons, Student's *t*-test was performed. There was no significant difference about the general data between the two groups (Table 1).

The following is the comparison of the perioperative period between the two groups: In the ESD group, the postoperative pathological examination of 2 cases could not determine whether there was tumor residual at the cutting edge due to electrocoagulation and cauterization, and the complete tumor resection rate was 90.0% (18/20). A total of 5 cases presented perforation during ESD surgery, 3 cases underwent surgical treatment, and the remaining 2 were successfully sealed with titanium clips. Postoperative fever occurred in 2 cases during ESD surgery, which were considered abdominal infection, and improved after anti-infective treatment, all of these were perforation patients. In the LECD group, the postoperative pathology of 25 cases showed complete capsules, no major intraoperative bleeding, perforation, and no postoperative complications such as bleeding and infection. There were no significant differences about the operation time, intraoperative bleeding, postoperative bleeding, and postoperative infection between the two groups (Tables 2 and 3). But the LECD group had a lower intraoperative perforation rate than the ESD group (0 vs. 25%, *p* < 0.01) and had some advantages of shorter postoperative indwelling gastric tube (1.04 ± 0.98 vs. 2.85 ± 0.24, *p* < 0.01), earlier postoperative eating (1.96 ± 0.98 vs. 3.50 ± 1.15, *p* < 0.01), and shorter average postoperative hospital stay (3.28 ± 0.94 vs. 7.85 ± 1.18, *p* < 0.01) (Tables 2 and 3).

### 3.2. Consumable Usage

For LECD surgical procedures, disposable tumor-related consumable materials, including one electrical hooks, one PDS thread or v-lock thread for suturing serosal surface wounds, and one endoscopic needle injection, were used under laparoscopy. The total cost of consumable materials was about 2000-2500 yuan. For ESD surgical procedures, ceramic knife and endoscopic closure system for closed mucosa were used, with a total cost of about 10,000 yuan. As for the previous wedge-shaped resection, it needs to use the linear laparoscopic cut stapler, at least including two space bookings and one gun barrel. And the total cost of the consumable materials is about 15,000 yuan. LECD surgery has tremendous advantages in financial burden for patients for the treatment of small mesenchymal tumors.

### 3.3. Pathological Examination of Tumor Samples

**Figure 3:**
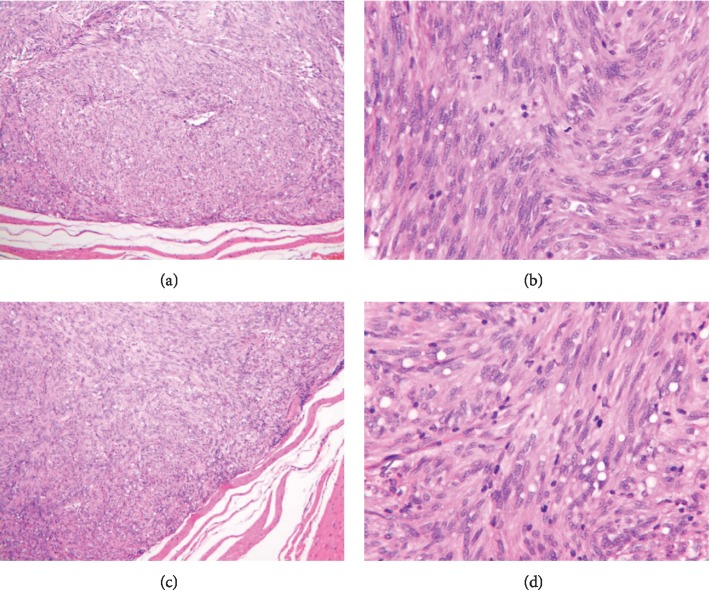
Postoperative pathology confirmed the presence of gastric stromal tumor, with complete envelope in all patients. (a, b) Tumor with complete capsule (HE ×100). (c, d) Mitotic counts (HE ×400).

All samples were gastric stromal tumors in LECD and ESD groups. In LECD group, according to the NIH risk classification, 19 cases were with very low risk GISTs, 4 cases were with low risk, and two cases with intermediate risk GISTs (Figure 3). In ESD group, 15 cases were with very low risk GISTs and 5 cases were with low risk.

### 3.4. Follow-Up Results

The follow-up was complete in all patients with two groups. The median follow-up time of ESD group and LECD group was 24 (12-42) months and 23 (12-40) months, respectively. No recurrence or metastasis cases were found between the two groups during the follow-up period, and there were no cases of death due to gastric GISTs. At the same time, in the previous cases of laparoscopic wedge resection and ESD gastric stromal tumor, there were scars on the mucosal surface when the gastroscope was reviewed. Since LECD patients did not damage the mucosal surface, tumor recurrence in situ could be better observed by postoperative gastroscopy follow-up.

## 4. Discussion

Epidemiological studies have confirmed that the incidence of small mesenchymal tumors in the stomach is much higher than that of interstitial tumors [2]. Nevertheless, whether gastric interstitial tumors should be resected is still a matter of controversy. Some studies have indicated that surgical resection increases trauma and the risk of implantation metastasis and limits the effectiveness of surgery in patients with small gastric stromal tumors and especially in those that show no risk factors of primary gastric stromal tumor under endoscopic ultrasonography. This is because this type of tumors often has benign clinical course [4], so they are not recommended for surgical resection and tend to be more closely followed up. Over recent years, however, some studies have shown that tumor size, even though related to dangerous degree classification, cannot be used to completely decide the prognosis and the malignant degree of stromal tumor. In addition, tumor recurrence and metastasis can occur in smaller stromal tumor cases [5, 6]. Therefore, even small mesenchymal tumors, once diagnosed by preoperative ultrasound endoscopy, need to be surgically resected [7]. Satisfactory R0 resection is useful for making the reasonable assessment of the small mesenchymal tumor malignancy according to the postoperative pathology and also to avoid the risk of recurrence and metastasis of small mesenchymal tumors. Over recent years, more and more clinicians have started to pay attention on how to guarantee the complete capsule in gastric stromal tumor resection (R0 resection) and to reduce the burden and surgical trauma of the patient. In addition, patients receive real benefit from the perspective of rapid recovery and minimally invasive surgery.

At present, the minimally invasive surgical methods for resection of gastric interstitial tumor include endoscopic surgery, laparoscopic surgery, and laparoscopic and endoscopic cooperative surgery [8]. Since it does not require the lymph node dissection, currently, the main method for laparoscopic surgery is the laparoscopic wedge resections. Compared with open surgery, laparoscopic wedge resection of gastric stromal tumors has shown to be safe and effective. Yet, this procedure is limited application in small mesenchymal tumors, especially for tumors that are less than 1 cm in size. On the one hand, laparoscopic localization of small stromal tumors is difficult; on the other hand, laparoscopic wedge resection requires the use of a section-closure device, which inevitably removes some healthy gastric tissue and damages the mucosa. Also, laparoscopic wedge resection is especially difficult in the gastroesophageal junction and the gastric pylorus and is prone to cause lumen stenosis. Consequently, laparoscopic wedge resection appears suitable for the appropriate cases, for example, patients with tumors larger than 2 cm or patients with tumors located in the gastric body [9]. The main advantages of endoscopic surgery are the fact that the pneumoperitoneum is not required and reduced the risk of general anesthesia. It is a good choice for advanced age and small gastric mesenchymoma [10]. The low complete cutting rate and the risk of perforation are the main restrictions of endoscopic surgery. Endoscopic full-thickness resection technology can effectively improve R0 resection; however, this technique needs to be performed by highly skilled operators and still cannot avoid big active perforation; this procedure brings the risk of abdominal cavity metastasis [11].

Laparoscopic and endoscopic cooperative surgery (LECS), which was first applied to gastric mesenchymoma resection, can be used to avoid the deficiency of single application of endoscopic surgical techniques and laparoscopic surgical technique [12]. Nevertheless, the conventional LECS procedure has the potential risk of gastric contents or tumor cells spilling into the abdominal cavity since the gastric wall has to be opened during the procedure [13, 14]. To avoid this problem, some modified LECS procedures have been investigated, such as inverted LECS [15], a combination of laparoscopic and endoscopic approaches to neoplasia with a nonexposure technique (CLEAN-NET) [16], nonexposed endoscopic wall-inversion surgery (NEWS) [17, 18], endoscopic full-thickness resection [19], and nonexposure laparoscopic and endoscopic cooperative surgery (closed-LECS) [20]. Although there are few reports on LECS, surgical safety and therapeutic effect on the tumor still require further evaluation. The existing studies have indicated that LECS operation is a very suitable surgical method for the resection of small mesenchymal tumors [21].

Several studies have reported that surgical procedures for gastric stromal tumors mentioned above, including combinations of two endoscopic procedures, may cause damage to the mucosa; thus, a postoperative gastric decompression is required for 2~10 d [8, 10]. In the present study, a novel procedure of laparoscopic surgery combined with endoscopic resection of small gastric stromal tumors was proposed in the light of anatomical features and biological behavior of small gastric stromal tumors and on the premise of achieving radical resection. Stromal tumors are located in the muscularis propria layer of the stomach wall, and the pathological pseudocapsule is actually a natural barrier between the gastric epithelium and the tumor cell; thus, it is necessary to ensure a complete and nondestructive level in radical surgery. However, the false capsule is extremely thin in natural condition, which is difficult to be removed from the submucosa without intervention, to ensure the integrity of the false capsule. The surgical design of this study was that through endoscopic localization, submucosal puncture was performed followed by water injection, so that the colored water cushion between the small stromal tumor and submucosa might be formed, which was more conducive in accurately locating and opening the serosa of interstitial tumor guided by the laparoscope and in removing the submucosal tumor under the submucosa, so as to protect the epithelial layer. Simultaneously, due to the existence of submucosal water cushion, it could effectively ensure the integrity of the false capsule during the excision process.

In this study, in the 25 cases who had undergone laparoscopic resection of small gastric stromal tumors, no gastric tube was placed after operation, and the liquid diet was recovered at 1-2 days postoperatively. The patients were discharged within 3 d after operation. Compared with conventional laparoscopic endoscopy combined with wedge resection of gastric stromal tumors, this approach leads to satisfactory short-term outcomes and meets the idea of minimally invasive surgery and rapid recovery. We compared ESD-treated small gastric GISTs in the same period and found that the LECD group had a lower intraoperative perforation rate than the ESD group and had some advantages of shorter postoperative indwelling gastric tube, eating earlier, and shorter average postoperative hospital stay. Meanwhile, for LECD group patients, the cost of tumor resection-related consumables was significantly lower than that for ESD and laparoscopic wedge resection group patients. This may be related to the high cost of disposable consumables for endoscopy in China, but it also proves the significance of LECD's further promotion in China.

Nevertheless, as a new surgical technique, LECD's safety and therapeutic effect needs to be further evaluated. In addition, the incidence of small gastric stromal tumors is high, and the origin, depth, layer, and size of the tumors directly determine the degree of difficulty in complete resection. Although the current cases have shown the good clinical effect of the procedure, how to choose the appropriate cases to carry out this new operation still needs further research and discussion.

## Figures and Tables

**Table 1:** Comparison of general data between LECD group and ESD group.

	LECD group	ESD group	*p* value
Age (y)	60.12 ± 9.24	58.25 ± 9.86	0.52
Sex			0.84
Male	12	9	
Female	13	11	
BMI (kg/m^2^)	22.52 ± 2.41	22.46 ± 2.36	0.94
Tumor diameter (cm)	1.38 ± 0.50	1.33 ± 0.41	0.7
Tumor location in the stomach			0.28
Cardia	0	2	
Fundus	11	8	
Antrum	4	1	
Body	10	9	
NIH risk classification			0.34
Very low risk	19	15	
Low risk	4	5	
Intermediate risk	2	0	
High risk	0	0	

**Table 2:** Comparison of perioperative data between LECD group and ESD group.

Observational index	LECD group (*n* = 25)	ESD group (*n* = 20)	*p* value
Complete tumor capsule	25/25	18/20 (90%)	0.11
Operation time (min)	80.76 ± 13.86	84.05 ± 15.33	0.45
Postoperative indwelling gastric tube (d)	1.04 ± 0.98	2.85 ± 0.24	<0.01
Postoperative eating time (d)	1.96 ± 0.98	3.50 ± 1.15	<0.01
Average hospital stay (d)	3.44 ± 1.00	7.85 ± 1.18	<0.01

**Table 3:** Comparison of postoperative complications between LECD group and ESD group.

Observational index	LECD group (*n* = 25)	ESD group (*n* = 20)	*p* value
Major intraoperative bleeding	0/25	1/20 (5%)	0.26
Postoperative bleeding	0/25	2/20 (10%)	0.11
Perforation	0/25	5/20 (25%)	<0.01
Postoperative infection	0/25	2/20 (10%)	0.11

## Data Availability

The authors declare that the data underlying the findings of this manuscript can be shared to allow researchers.

## Abbreviations

GIST: Gastrointestinal stromal tumor
LECD: Laparoscopic and endoscopic cooperative dissection.
